# Reduced Histidine Metabolism Is Associated with Early Allograft Dysfunction Following Liver Transplantation

**DOI:** 10.3390/metabo16070449

**Published:** 2026-06-26

**Authors:** Alissa M. Cutrone, Thomas Agius, Sofia Baptista, Eleonore Baughan, Korkut Uygun, Alban Longchamp, Heidi Yeh

**Affiliations:** 1 Center for Transplantation Sciences, Massachusetts General Hospital, Harvard Medical School, Boston, MA 02114, USA; acutrone@mgh.harvard.edu (A.M.C.); sofia.baptista@umassmed.edu (S.B.); eleonore.baughan@mountsinai.org (E.B.); alongchamp@mgh.harvard.edu (A.L.); 2 Center for Engineering in Medicine and Surgery, Department of Surgery, Massachusetts General Hospital, Harvard Medical School, Boston, MA 02114, USA; thomas.agius@hest.ethz.ch (T.A.); kuygun@mgh.harvard.edu (K.U.); 3 Shriners Children’s—Boston, Boston, MA 02114, USA

**Keywords:** liver transplantation, early allograft dysfunction, histidine metabolism, ischemia-reperfusion injury, graft resilience, biomarkers, machine perfusion

## Abstract

**Highlights:**

**What are the main findings?**

- Untargeted metabolomics identified histidine metabolism as the dominant enriched pathway associated with immediate graft function following liver transplantation.
- Trans-urocanate emerged as the strongest discriminatory metabolite associated with early allograft dysfunction, demonstrating 80% sensitivity and 80% specificity.

**What are the implications of the main findings?**

- Histidine metabolism may represent a previously unrecognized signature of liver graft resilience to ischemia-reperfusion injury.
- Metabolomic profiling during the perfusion era may support biomarker-guided liver viability assessment and future metabolic optimization strategies.

**Abstract:**

**Background/Objectives**: Early allograft dysfunction (EAD) is a common complication after liver transplantation and is associated with inferior graft survival. While normothermic machine perfusion (NMP) has reduced EAD incidence, the prediction of early graft performance prior to implantation remains elusive. We aimed to correlate the peri-transplant energetic and metabolic profile of liver grafts with post-transplant outcome in a cohort that included grafts preserved with NMP. **Methods**: Sequential biopsies were taken from 20 transplanted livers (10 immediate graft function [IGF] and 10 EAD), preserved by either static cold storage or NMP. Samples were collected immediately prior to implantation and 30 min after hepatic arterial reperfusion. Untargeted liquid chromatography-mass spectrometry was performed, and energy charge was calculated as (ATP + 1/2 ADP)/(ATP + ADP + AMP). Univariate and receiver operating characteristic analysis identified metabolites correlated with EAD and assessed predictive accuracy. **Results**: Hepatic concentrations of adenine nucleotides and calculated energy charge did not differ between outcome groups either before implantation or after reperfusion. In contrast, trans-urocanate was significantly enriched in IGF livers across both time points, and additional histidine catabolism pathway metabolites were preferentially increased in IGF grafts. Trans-urocanate demonstrated discriminatory performance for EAD with 80% sensitivity and 80% specificity, confirmed as the single strongest predictive feature among >1600 detected metabolites. **Conclusions**: These data identify histidine catabolism as a novel metabolic pathway associated with early graft function and a potential indicator of allograft resilience to ischemia-reperfusion injury. Integration of histidine pathway metabolites into perfusion-era viability assessment may serve as a discriminative biomarker of EAD and support future metabolite-guided graft optimization strategies.

## 1. Introduction

Early allograft dysfunction (EAD) occurs in up to 20–40% of liver transplants despite advances in donor graft procurement, preservation, and perioperative management [1,2]. EAD is associated with increased intensive care utilization, prolonged hospital length of stay, and inferior short and long-term outcomes, including reduced graft survival and increased mortality [3,4]. Extended-criteria donors, including donation after circulatory death (DCD), steatotic grafts, and older grafts, are increasingly used in transplant programs despite their elevated risk for EAD [5,6], highlighting a critical unmet need for biomarkers that can predict graft performance [7,8].

Normothermic machine perfusion (NMP) has emerged as a transformative preservation technology that not only extends preservation time but also enables real-time functional assessment of donor livers prior to transplantation [9,10]. Current viability assessment during NMP relies mostly on lactate clearance, bile production, and vascular flows [11], all metrics based on the central role of adenosine triphosphate (ATP) depletion and mitochondrial failure during ischemia-reperfusion injury (IRI) [12,13]. Previous mechanistic studies have demonstrated that adenine nucleotide content and hepatic energy charge were relevant molecular indicators of organ viability [14,15]. Because EAD can only be diagnosed after transplantation, biomarkers measurable during preservation are needed to identify high-risk grafts before implantation. Identifying additional metabolic metrics during NMP may enable real-time discrimination between grafts that will develop EAD and those that will achieve immediate graft function (IGF), creating opportunities for earlier risk stratification and potential targeted interventions in the modern perfusion era.

Prior metabolomic studies have demonstrated that amino acid metabolism, redox buffering pathways, and mitochondrial substrate utilization are dynamically altered during machine perfusion and transplantation [16,17]. In this study, we performed untargeted metabolomic profiling of human liver allograft biopsies obtained immediately prior to implantation and following hepatic arterial reperfusion in livers preserved with static cold storage (SCS) or having undergone NMP. Our goal was to identify molecular signatures associated with early graft outcome that could be targeted for intervention during machine perfusion. Specifically, we examined traditional energy metrics and alternative metabolic pathways to better discriminate between grafts that developed EAD and those that achieved IGF.

## 2. Materials and Methods

### 2.1. Study Population

Adult candidate recipients (aged 18–75 years) for deceased donor liver transplantation were included in this study following informed consent. To identify relevant metabolites, we selected 10 control patients with IGF and 10 patients who suffered from EAD from a larger cohort of patients who underwent transplant between February 2022 and November 2023. Selection was done by chronological order of transplantation and with the goal of donor-matching between groups for sex and donor type (Table 1). In each group, half of the grafts were placed on NMP, while the other half was transported on SCS. Exclusion criteria included grafts from donors with HIV-1 seropositivity and recipients with acute liver failure requiring emergent transplantation.

Patients received perioperative standards of care for liver transplantation. Post-transplant recipient liver function tests, international normalized ratio, ICU length of stay (LOS), pressor use, HCV status, and recipient warm ischemic time (WIT) were determined by retrospective chart review. Donor age, gender, donor type (DCD vs. donation after brain death [DBD]), and cold ischemic time (CIT) were determined by retrospective review of the recipient medical chart, which had information transferred from the donor packet accompanying the donor organ. EAD was defined by the presence of at least one of the following criteria: bilirubin > 10 mg/dL on post-op day 7 (POD7), INR > 1.6 on POD7, or ALT or AST > 2000 IU/L within the first 7 days post-op (1). The Mass General Brigham Investigational Review Board approved this clinical study (IRB protocol #2013P000872).

### 2.2. Surgical and Biopsy Procedure

All donor livers for transplantation were obtained following the standard procurement procedure for DBD or DCD. None of the livers underwent normothermic regional perfusion (NRP). Livers were cold-flushed in situ with the University of Wisconsin (UW) solution. Livers undergoing SCS were transported in UW preservation solution on ice. Livers undergoing NMP were perfused using the Liver Organ Care System (TransMedics Inc., Andover, MA, USA) according to the manufacturer’s instructions. Livers were flushed through the portal vein with lactated Ringer’s–albumin solution immediately before implantation. Arterial anastomoses and reperfusion were performed separately and after portal reperfusion. A maximum of 2 needle biopsies were taken at sequential time points: immediately before implantation (out of ice [OOI]) and 30 min after hepatic artery reperfusion (HAR). Samples were immediately (<10 s) flash-frozen in the operating room using liquid nitrogen and stored at −80 °C until further analysis.

### 2.3. Untargeted Metabolomic Analysis

Tissue samples were homogenized, centrifuged, and the supernatant was analyzed by Hydrophilic Interaction Liquid Chromatography coupled to tandem mass spectrometry (HILIC-MS/MS). Proteins were extracted in Tris-HCl buffer with protease/phosphatase inhibitors and quantified by BCA Protein Assay Kit (Thermo Scientific, Waltham, MA, USA). HILIC-MS/MS was performed in both positive and negative ionization modes using an Agilent 6495 QgQ system with a 1290 UHPLC system (Agilent, Santa Clara, CA, USA). Data acquisition and metabolite quantification were performed using Agilent MassHunter software (B.07.00) based on Extracted Ion Chromatogram areas for multiple reaction monitoring (MRM) transitions. Peak areas were analyzed in R with drift correction and noise filtering (CV > 30%) performed using MRM PROBS software. Relative metabolite abundances were normalized prior to downstream analyses. Energy charge was calculated as [ATP + ADP × 0.5]/[ATP + ADP + AMP].

Multivariate analyses were performed using MetaboAnalyst. Partial least squares-discriminant analysis (PLS-DA) was used to assess metabolic separation between grafts with IGF and EAD, and PLS-DA model performance was assessed using repeated cross-validation. Variable importance in projection (VIP) scores were calculated to identify metabolites contributing most strongly to group discrimination. Metabolite set enrichment analysis was performed using pathway-associated metabolite libraries to identify pathways associated with graft outcome. Receiver operating characteristic (ROC) analyses were performed to evaluate the discriminatory performance of candidate metabolites for the prediction of EAD. Monte Carlo cross-validation was used for multivariate ROC modeling, classifier evaluation, and feature ranking.

### 2.4. Statistical Analysis

Continuous variables were compared using Mann–Whitney U tests and categorical variables using Χ^2^ tests, as appropriate. Subgroup comparisons were performed using Kruskal–Wallis testing with Dunn’s multiple-comparisons correction. Multivariable logistic regression was performed for select variables, with odds ratios (OR) and 95% confidence intervals (CI) reported. Statistical analyses were performed using R version 4.4.2 (31 October 2024) and GraphPad Prism version 11.0.2 (GraphPad Software, San Diego, CA, USA). Data are presented as median (IQR) unless otherwise noted.

## 3. Results

### 3.1. Donor and Recipient Characteristics

Donor and recipient characteristics are presented in Table 1. A total of 20 transplanted livers were analyzed, including 10 grafts that developed EAD and 10 with IGF. Donor characteristics were similar between groups, including donor age, sex, donor type, and total WIT. DBD and DCD grafts were equally represented between outcome groups. CIT trended longer in the EAD group, but did not reach statistical significance. Recipient age and Model for End-Stage Liver Disease (MELD) score were similar between groups. However, the recipient body mass index (BMI) was significantly higher in the EAD group (median 31.6 vs. 25.6 kg/m^2^, *p* = 0.008).

### 3.2. Histidine Metabolism Is the Dominant Enriched Metabolic Pathway Associated with Immediate Graft Function

**Figure 1:**
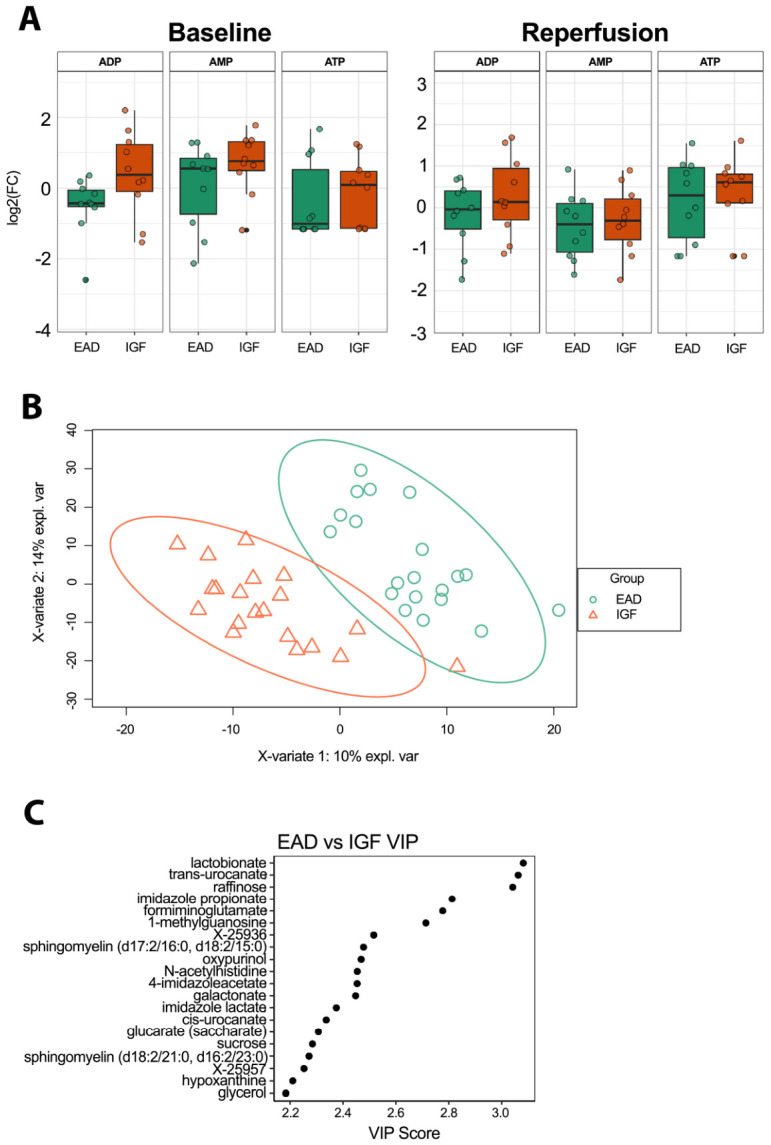
**Untargeted metabolomic profiling identifies global metabolic separation between IGF and EAD grafts despite similar hepatic energy charge.** (**A**) Fold change in hepatic ATP, ADP, and AMP concentrations before and after reperfusion for IGF (in red) and EAD (in green) grafts. (**B**) PLS-DA of untargeted metabolomic profiles demonstrating metabolic separation between IGF (red) and EAD (green) grafts. (**C**) VIP scores identifying metabolites contributing most strongly to group discrimination in the PLS-DA, including trans-urocanate and lactobionate. Baseline (pre-implantation) and reperfusion (post-HAR) biopsies were obtained from 10 EAD and 10 IGF grafts.

Direct quantification of adenine nucleotides demonstrated no significant differences in ATP, ADP, and AMP concentrations or calculated energy charge between IGF and EAD grafts (Figure 1A). Calculated hepatic energy charge demonstrated substantial overlap between groups both before and after HAR, therefore failing to discriminate graft outcome in this cohort. Despite the absence of differences in conventional energetic metrics, untargeted metabolomic profiling revealed distinct metabolic separation between IGF and EAD grafts by PLS-DA (Figure 1B). Cross-validation analysis also demonstrated stable classifier performance across multiple feature sets, with optimal predictive accuracy achieved using approximately 15 metabolite features. VIP analysis identified lactobionate and trans-urocanate among the highest-ranking discriminatory metabolites contributing to separation between outcome groups (Figure 1C).

**Figure 2:**
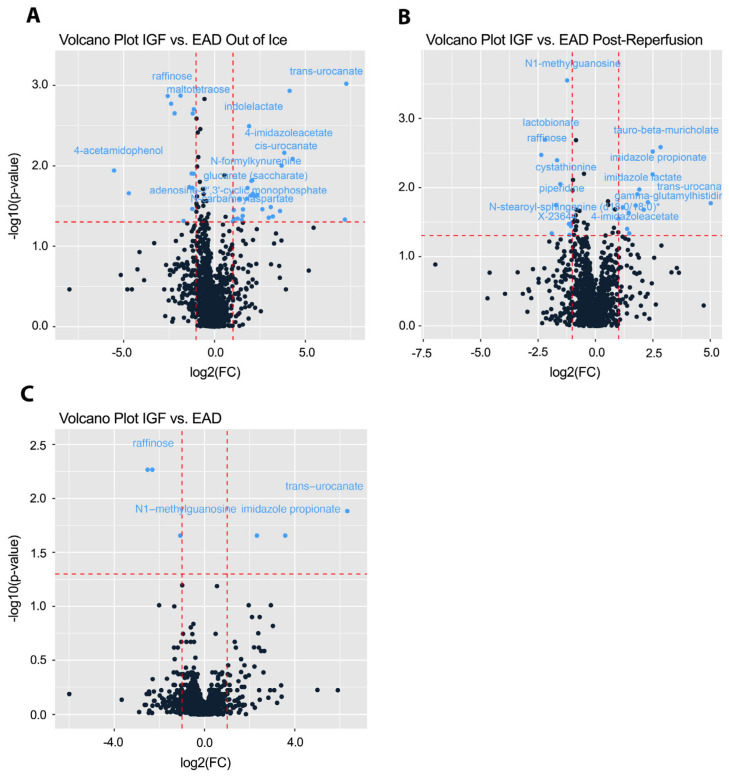
**Trans-urocanate is enriched in IGF grafts across reperfusion states.** Differential metabolite abundance in biopsies pre-implantation (**A**), post-reperfusion (**B**), and combined timepoint analyses (**C**), showing significant enrichment of trans-urocanate in IGF grafts.

Univariate metabolomic analysis identified trans-urocanate as the most significantly enriched metabolite in IGF livers. This enrichment was observed across pre-reperfusion (Figure 2A), post-reperfusion (Figure 2B), and when analyzing the time points together (Figure 2C). Seven of the top 20 VIP-ranked metabolites in IGF livers belonged to the histidine degradation pathway, including trans-urocanate, imidazole propionate, formiminoglutamate, N-acetylhistidine, 4-imidazoleacetate, imidazole lactate, and cis-urocanate. Non-histidine metabolites, such as lactobionate, demonstrated weaker and less consistent discriminatory performance.

**Figure 3:**
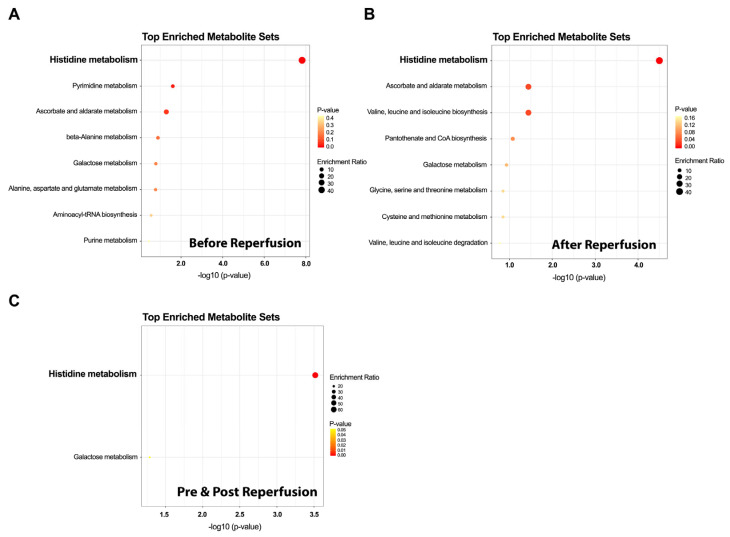
**Metabolite set enrichment analysis identifies coordinated activation of histidine metabolism in grafts with immediate graft function.** Pathway enrichment analysis performed in MetaboAnalyst using untargeted metabolomic profiles from liver graft biopsies. Histidine metabolism emerged as the most significantly enriched metabolite set in IGF vs. EAD grafts, both before reperfusion (**A**), after reperfusion (**B**), and in combined analyses (**C**). Bubble size indicates enrichment ratio and bubble color corresponds to pathway significance (−log10 *p*-value).

To determine whether coordinated metabolic pathways distinguish graft outcomes, metabolite set enrichment analysis was performed using MetaboAnalyst. Histidine metabolism emerged as the dominant enriched metabolic pathway associated with IGF across timepoints (Figure 3A–C). Additional enriched pathways demonstrated substantially lower enrichment ratios and statistical significance, supporting histidine metabolism as the principal metabolic signature associated with IGF.

### 3.3. Low Trans-Urocanate Associated with Early Allograft Dysfunction

**Figure 4:**
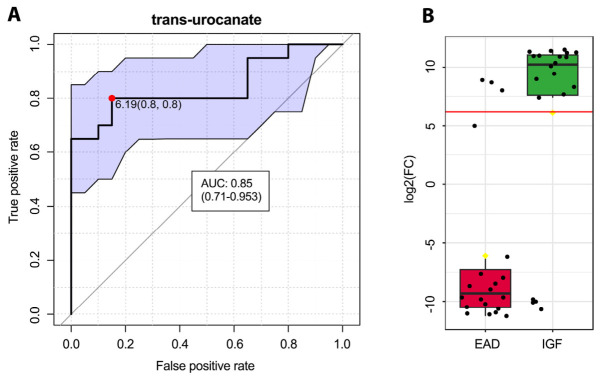
**Trans-urocanate demonstrates strong discriminatory performance for EAD.** (**A**) ROC curve demonstrating the ability of hepatic trans-urocanate to discriminate EAD from IGF grafts with 80% sensitivity and 80% specificity. (**B**) Fold change in tissue concentrations of trans-urocanate in IGF vs. EAD grafts, with significantly reduced levels in grafts developing EAD.

ROC analysis demonstrated that low trans-urocanate was able to predict EAD with 80% sensitivity and 80% specificity (Figure 4A). Tissue concentrations of trans-urocanate were significantly reduced in EAD grafts compared with IGF grafts (Figure 4B), consistent with metabolomic enrichment analyses observed across reperfusion states.

To evaluate the potential influence of confounding variables such as preservation modality and recipient BMI, exploratory subgroup and multivariable analyses were performed. Subgroup analyses revealed that trans-urocanate abundance did not differ between grafts preserved with NMP and SCS (*p* = 0.853). When stratified by both preservation modality and graft outcome (EAD-NMP, EAD-SCS, IGF-NMP, and IGF-SCS), pairwise comparisons demonstrated no significant differences between NMP and SCS grafts within either outcome group (Figure S1). Additionally, multivariable logistic regression analyses revealed that while higher BMI was associated with increased odds of EAD (OR 1.37, 95% CI 1.03–2.16), higher trans-urocanate remained associated with reduced odds of EAD (OR 0.86, 95% CI 0.72–0.97).

**Figure 5:**
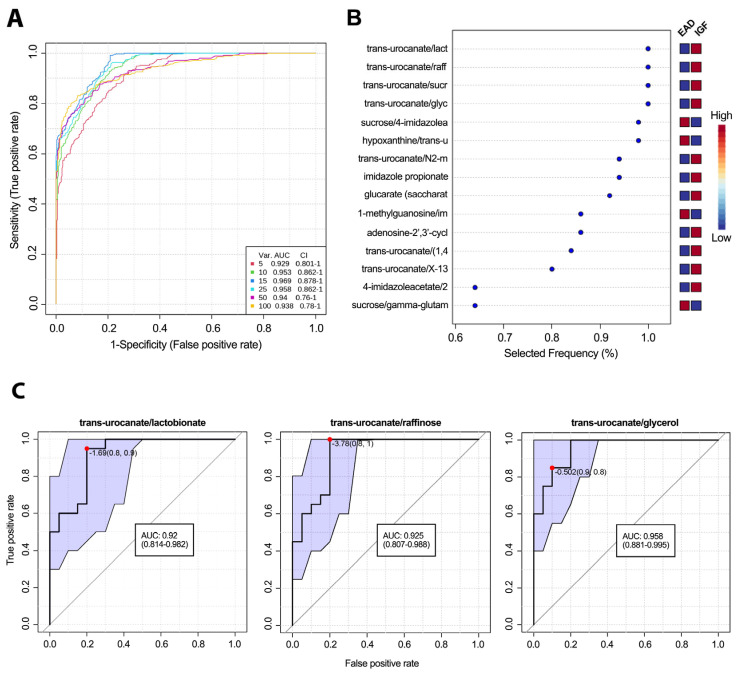
**Multivariate metabolomic classifiers identify trans-urocanate as the dominant predictive feature associated with graft outcome.** (**A**) Monte Carlo cross-validation analysis ROC curves demonstrating predictive accuracy of multivariate metabolites classifiers across increasing feature-set sizes. (**B**) Feature importance ranking showing trans-urocanate as the highest-ranking contributor to classifier performance across multivariate models. (**C**) ROC performance of the three top-performing two-metabolite classifiers, demonstrating only modest improvement in discriminatory performance with additional metabolites beyond trans-urocanate.

To further evaluate the discriminatory performance of metabolomic features associated with graft outcome, Monte Carlo cross-validated multivariate ROC modeling was performed. Composite metabolite classifiers demonstrated robust discrimination between IGF and EAD grafts (Figure 5A). Across all multivariate models, trans-urocanate consistently emerged as the most dominant predictive feature (Figure 5B). Notably, inclusion of additional metabolites provided only modest improvement in discriminatory performance, relative to trans-urocanate alone, as demonstrated by the top-performing two-metabolite classifiers (Figure 5C).

## 4. Discussion

This study identifies histidine metabolism, specifically trans-urocanate, as a previously unrecognized metabolic discriminator of early liver allograft function. Across unbiased metabolic profiling, multiple histidine-derived intermediates were enriched in grafts that achieve immediate function, with low trans-urocanate emerging as the single strongest predictive feature of EAD. These findings identify histidine catabolism as a previously underappreciated pathway that may contribute to hepatocellular resilience during IRI. Among all detected metabolites, trans-urocanate demonstrated the most dominant and most consistent association with IGF, predicting EAD with 80% sensitivity and 80% specificity. Notably, its elevation was observed both before implantation and after reperfusion, suggesting that it reflects an intrinsic metabolic state of the graft rather than a secondary response to reperfusion injury.

Importantly, the metabolite signature associated with graft function was not restricted to a single metabolite. Of the top 20 VIP-ranked features, seven belonged to the histidine degradation pathway, including multiple other imidazole derivatives. This coordinated enrichment strongly supports a pathway-level biological effect, rather than a stochastic statistical association. The presence of both upstream and downstream histidine metabolites suggests that overall pathway flux, rather than isolated accumulation, is what differentiates protected grafts from those that fail early. This raises the question of whether impaired activation of histidine catabolism is a metabolic vulnerability state that predisposes grafts to reperfusion injury.

Histidase (histidine ammonia-lyase, encoded by HAL), the rate-limiting enzyme in histidine catabolism, catalyzes the conversion of histidine to urocanate and is predominantly expressed in the liver, making hepatic tissue uniquely positioned for histidine degradation and urocanate production [18,19]. Histidine catabolism produces imidazole-containing intermediates, including trans-urocanate, that possess antioxidant, proton-buffering, and redox-modulating properties [20,21]. These characteristics are particularly relevant during the reperfusion phase of transplantation, when oxygen reintroduction drives mitochondrial reactive oxygen species (ROS) generation and oxidative damage [22]. Imidazole-containing intermediates modulate oxidative stress and inflammatory responses in experimental hepatic IRI models [23,24], supporting a potential link between histidine metabolism and graft recovery following transplantation. Elevated histidine flux in the IGF livers may enhance intracellular hepatic buffering capacity, stabilizing cytosolic and mitochondrial pH during abrupt metabolic transitions, while its derivatives scavenge ROS and limit oxidative damage to mitochondrial membranes and respiratory chain proteins. However, antioxidant buffering may represent only one of several mechanisms underlying the observed association between histidine pathway metabolites and graft outcome. Histidine catabolism is also integrated with broader aspects of hepatic metabolic function, including amino acid homeostasis, nitrogen handling, and one-carbon metabolism through intermediates such as formiminoglutamate [21]. Consequently, reduced abundance of trans-urocanate and related metabolites may therefore reflect a more global impaired hepatocellular metabolic competence than an isolated deficiency. Altered HAL activity, disrupted amino acid utilization, impaired mitochondrial recovery, or generalized metabolic dysfunction following IRI could all contribute to the metabolomic signature observed in EAD grafts. While the mechanistic relationship between histidine metabolism and graft function remains incompletely understood, these findings suggest that histidine pathway metabolites may serve as markers of preserved hepatic metabolic function and resilience to IRI, and provide a basis for improvement of organ preservation outcomes via histidine-based strategies.

Recipient BMI was significantly higher in the EAD group, consistent with known associations between obesity, mitochondrial stress, impaired antioxidant capacity, and heightened susceptibility to IRI and poor transplant outcome [25,26]. Obesity is also characterized by altered amino acid handling and diminished redox flexibility [27]. It is therefore plausible that grafts transplanted into recipients with higher BMI face a greater burden of oxidative and inflammatory injury, which may influence susceptibility to EAD. However, exploratory multivariable analysis suggested that the association between trans-urocanate abundance and graft outcome persisted after adjustment for recipient BMI, indicating that the observed histidine metabolism signal is unlikely to be explained solely by differences in recipient metabolic risk. Nevertheless, given the limited sample size, these findings should be interpreted cautiously and require validation in larger cohorts. Collectively, these observations, along with the reproducibility of the histidine signal across donors, preservation conditions, and time points, suggest that histidine metabolism more likely reflects a graft-intrinsic metabolic program associated with resilience to injury, rather than a surrogate of recipient metabolic risk.

Of note, a previous study found increased histidine metabolites in pre-transplant biopsies of EAD livers [28]. However, these were pre-preservation biopsies taken immediately after procurement, with only 30–40 min of CIT, so that very little ischemic and no reperfusion injury would have occurred. The profile of histidine metabolism observed in their samples, therefore, would not reflect changes during cold storage, NMP, or reperfusion. In contrast, our biopsies were obtained after preservation and at reperfusion, capturing the metabolic state of the graft at the time most relevant to IRI-mediated injury. The reversed directionality of histidine metabolite enrichment between the two studies may reflect the dynamic, time-dependent nature of histidine catabolism during the transplant process. Another potential explanation for the discrepant findings involves altered activity of histidase. Reduced HAL activity could theoretically limit conversion of histidine to urocanate and downstream metabolites. Whether IRI, prolonged cold storage, or machine perfusion directly influence HAL activity remains unknown and was not evaluated in the present study.

NMP has shifted the paradigm of graft evaluation from static risk estimation to dynamic functional interrogation. Current assessment tools emphasize global physiologic performance, with emerging molecular markers such as flavin mononucleotide (FMN) being validated for mitochondrial viability assessment during hypothermic machine perfusion [29]. Our findings suggest that histidine pathway metabolites may add a molecular layer of stress-resilience profiling that is preserved across different preservation strategies, identifying grafts that are biochemically equipped to withstand reperfusion. From a translational perspective, histidine metabolites are attractive biomarker candidates for both diagnostic and therapeutic purposes. Future studies should evaluate whether trans-urocanate and related metabolites can be measured in perfusate during NMP for non-invasive and real-time viability assessment, as well as exploring histidine supplementation or pathway priming as a way to actively enhance graft resilience prior to implantation.

However, this study has several limitations. The small cohort size, along with multiple preservation modalities, limited statistical power for subgroup analyses. For example, although donor type was balanced between outcome groups, additional donor variables associated with EAD risk, including donor hospital course, acid-base status, and liver enzyme profiles, were not incorporated into the present analysis and should be evaluated in larger cohorts. Given the exploratory nature of this untargeted metabolomic study, these findings should be considered hypothesis-generating and will require further validation. While coordinated pathway enrichment supports biologic plausibility, direct causal testing will require experimental manipulation of histidine flux during machine perfusion and transplantation. In addition, longitudinal outcome correlations beyond EAD remain to be investigated.

## 5. Conclusions

Histidine metabolism emerged as the dominant metabolic pathway associated with immediate graft function following liver transplantation. Among all detected metabolites, reduced trans-urocanate demonstrated the strongest association with early allograft dysfunction and may represent a biomarker of hepatic resilience to ischemia-reperfusion injury. These findings support the integration of metabolomic approaches into perfusion-era graft assessment and provide a rationale for future studies investigating histidine pathway metabolites as biomarkers and therapeutic targets for graft optimization. More broadly, they suggest a shift from assessing graft injury alone toward characterizing graft metabolic stress tolerance and provide a framework for exploring perfusion-based metabolic reprogramming strategies.

## Figures and Tables

**Table 1:** Donor and recipient demographics.

	IGF	EAD	*p*-Value
**DONOR**			
**Age**			
Median [IQR]	41.5 [24.8, 53.0]	51.0 [41.8, 59.3]	0.265
**Sex**			
Female	5 (50.0%)	3 (30.0%)	0.65
Male	5 (50.0%)	7 (70.0%)	
**Pump**			
No	5 (50.0%)	5 (50.0%)	1
Yes	5 (50.0%)	5 (50.0%)	
**Donor Type**			
DBD	6 (60.0%)	6 (60.0%)	1
DCD	4 (40.0%)	4 (40.0%)	
**Warm Ischemic Time**			
Median [IQR]	23.0 [22.5, 24.0]	28.0 [19.0, 30.0]	0.408
**Cold Ischemic Time**			
Median [IQR]	265.0 [150.0, 343.0]	422.0 [385.0, 454.0]	0.068
**RECIPIENT**			
**Age**			
Median [IQR]	59.5 [51.0, 63.5]	56.0 [48.0, 62.3]	0.692
**Sex**			
Female	5 (50.0%)	4 (40.0%)	1
Male	5 (50.0%)	6 (60.0%)	
**BMI**			
Median [IQR]	25.6 [24.7, 28.3]	31.6 [29.7, 36.8]	0.008
**MELD score**			
Median [IQR]	27.0 [22.8, 28.0]	27.5 [19.5, 30.5]	0.572

BMI = body mass index; DBD = donation after brain death; DCD = donation after circulatory death; IQR = interquartile range; MELD = Model for End-Stage Liver Disease.

## Data Availability

The raw data supporting the conclusions of this article will be made available by the authors on request.

## Supplementary Materials

The following supporting information can be downloaded at https://www.mdpi.com/article/10.3390/metabo16070449/s1, Figure S1: Exploratory subgroup analysis of trans-urocanate abundance by preservation modality and graft outcome. Normalized trans-urocanate abundance in EAD-NMP (*n* = 5), EAD-SCS (*n* = 5), IGF-NMP (*n* = 5), and IGF-SCS (*n* = 5) grafts. Points represent individual grafts; horizontal bars indicate median and interquartile range. Although an overall Kruskal–Wallis test was significant (*p* = 0.0267), no pairwise comparisons remained significant following Dunn’s multiple comparisons correction.

## Abbreviations

The following abbreviations are used in this manuscript:

ADP	adenosine diphosphate
AMP	adenosine monophosphate
ATP	adenosine triphosphate
DBD	donor/donation after brainstem death
DCD	donor/donation after circulatory death
EAD	early allograft dysfunction
FMN	flavin mononucleotide
HAL	histidine ammonia-lyase
HAR	hepatic arterial reperfusion
HMP	hypothermic machine perfusion
HTK-N	histidine-tryptophan-ketoglutarate n-acetyl-histidine
IGF	immediate graft function
IRI	ischemia-reperfusion injury
NADPH	nicotinamide adenine dinucleotide phosphate
NMP	normothermic machine perfusion
NRP	normothermic regional perfusion
PLS-DA	partial least squares discrimination analysis
ROC	receiver operating characteristic
ROS	reactive oxygen species
SCS	static cold storage
UW	University of Wisconsin
VIP	variable importance in projection
