# Supplementary material for: Reduced Histidine Metabolism Is Associated with Early Allograft Dysfunction Following Liver Transplantation
Source: Metabolites. 2026 Jun 26;16(7):449. doi: 10.3390/metabo16070449 (PMC13413880; doi:10.3390/metabo16070449)
Supplement: Supplementary file 1 [file metabolites-16-00449-s001.zip › metabolites-4355173-supplementary.pdf]

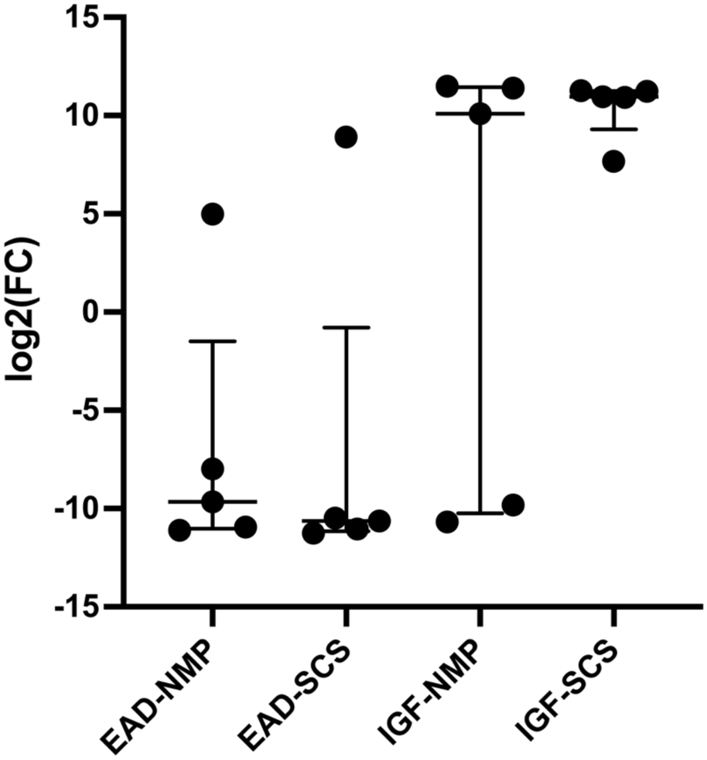

Supplementary Figure S1. Exploratory subgroup analysis of trans-urocanate abundance by preservation modality and graft outcome. Normalized trans-urocanate abundance in EAD-NMP (n=5), EAD-SCS (n=5), IGF-NMP (n=5) and IGF-SCS (n=5) grafts. Points represent individual grafts; horizontal bars indicate median and interquartile range. Although an overall Kruskall-Wallis test was significant (p=0.0267), no pairwise comparisons remained significant following Dunn's multiple comparisons correction.
